# Supplementary material for: Strategies of Advanced Airway Management in Out-of-Hospital Cardiac Arrest during Intra-Arrest Hypothermia: Insights from the PRINCESS Trial
Source: J Clin Med. 2022 Oct 28;11(21):6370. doi: 10.3390/jcm11216370 (PMC9654441; doi:10.3390/jcm11216370)
Supplement: Supplementary file 1 [file jcm-11-06370-s001.zip › Supplemental Figure S1.pdf]

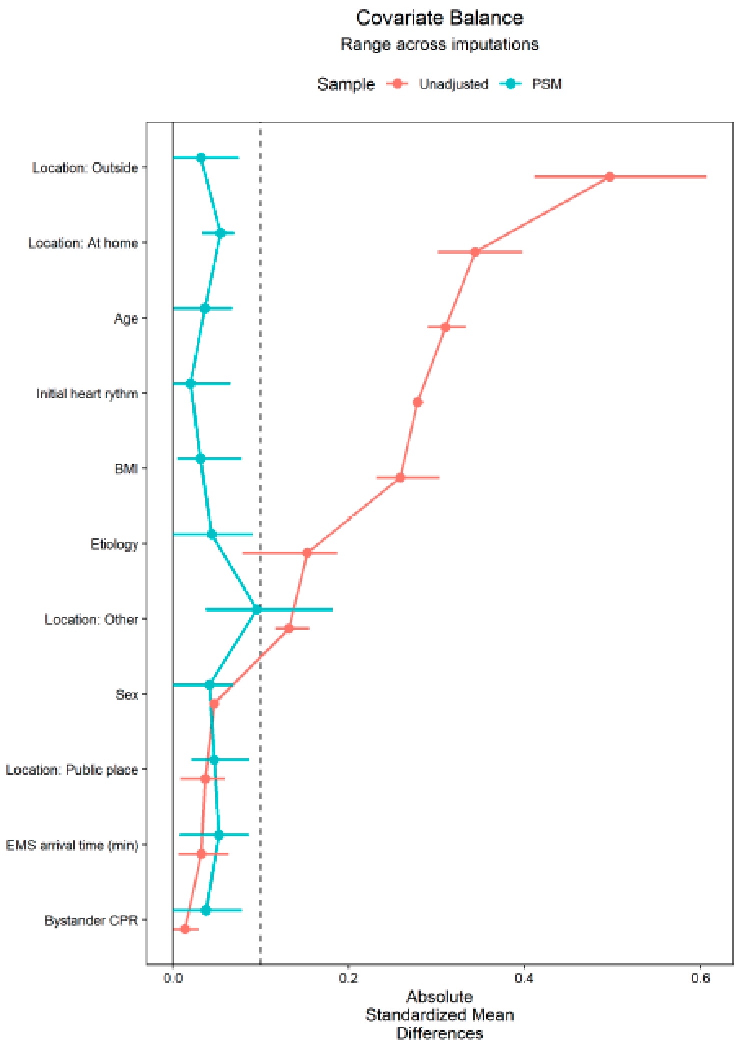

Supplemental Figure S1: Standardized mean differences before and after propensity score matching. Standardized Mean Differences (SMDs) at baseline (orange) and after propensity score matching (blue). The adjusted variables are displayed on the y-axis. SMD > 0,1 was considered to be indicative of a statistically significant group difference.
