# Supplementary material for: Strategies of Advanced Airway Management in Out-of-Hospital Cardiac Arrest during Intra-Arrest Hypothermia: Insights from the PRINCESS Trial
Source: J Clin Med. 2022 Oct 28;11(21):6370. doi: 10.3390/jcm11216370 (PMC9654441; doi:10.3390/jcm11216370)
Supplement: Supplementary file 1 [file jcm-11-06370-s001.zip › Supplemental Figure S2.pdf]

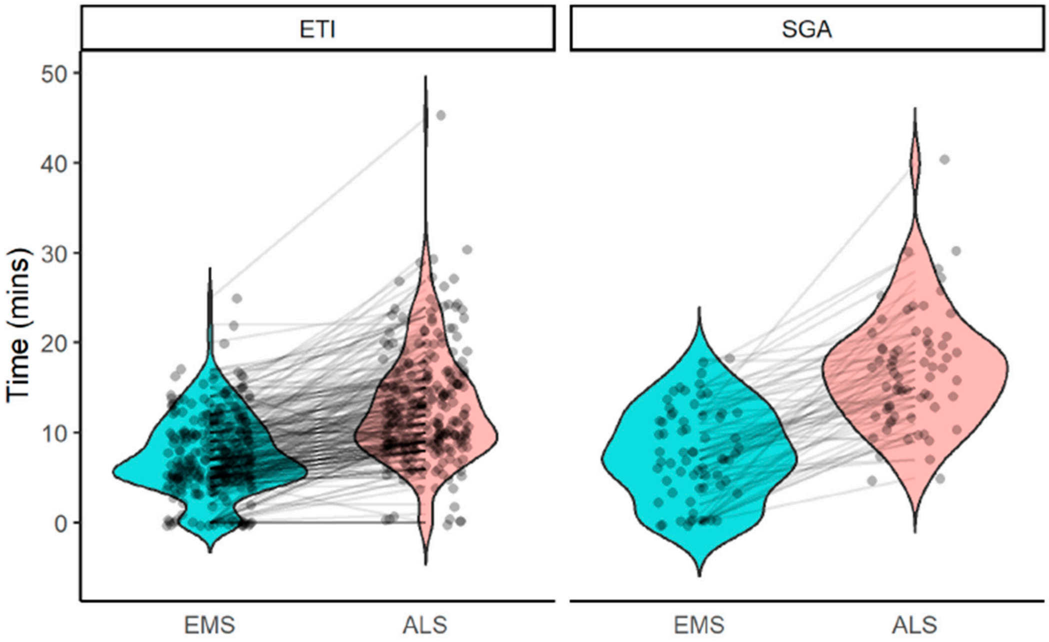

Supplemental Figure S2.

The median respone time in minutes for the first tier ambulance was 7 minutes (Interquartile range [IQR] 5-10) in the ETI group and 7.5 minutes (IQR 4-12) i the SGA group.

The median time for the second tier ALS vechicle was 12 minutes (IQR 9-16) in the ETI group versus 17 minutes (IQR 13-20) in the SGA group.
