# Supplementary material for: Strategies of Advanced Airway Management in Out-of-Hospital Cardiac Arrest during Intra-Arrest Hypothermia: Insights from the PRINCESS Trial
Source: J Clin Med. 2022 Oct 28;11(21):6370. doi: 10.3390/jcm11216370 (PMC9654441; doi:10.3390/jcm11216370)
Supplement: Supplementary file 1 [file jcm-11-06370-s001.zip › Supplemental Table S1.pdf]

**Variable**	**Missing**	**Proportion**
Location	36	11 %
Etiology	27	8 %
BMI	26	8 %
Bystander CPR	15	5 %
Age	8	2 %
EMS arrival time (min)	7	2 %
Initial heart rythm	1	0 %

Supplemental Table S1: Missing data for the predictor variables (where present). Abbreviations: BMI = Body mass index in units of kg/m^2, CPR = Cardiopulmonary resuscitation, EMS = Emergency medical services.
