# Supplementary material for: Strategies of Advanced Airway Management in Out-of-Hospital Cardiac Arrest during Intra-Arrest Hypothermia: Insights from the PRINCESS Trial
Source: J Clin Med. 2022 Oct 28;11(21):6370. doi: 10.3390/jcm11216370 (PMC9654441; doi:10.3390/jcm11216370)
Supplement: Supplementary file 1 [file jcm-11-06370-s001.zip › Supplemental Table S2.pdf]

	Median time in minutes (Q1 - Q3)			
**Outcome**	**Patients with favourable outcomes**	**Patients with unfavourable outcomes**	**OR (95% CI)**	**p value**
**Time until intra-arrest cooling**				
Survival	9 (6.00 - 13.00)	12 (8.00 - 18.00)	0.95 (0.91 - 0.99)	0.017
Sustained ROSC	10 (7.00 - 16.00)	12 (8.25 - 18.00)	0.98 (0.95 - 1.00)	0.088
CPC 1 - 2	9 (6.50 - 13.50)	12 (8.00 - 18.00)	0.96 (0.92 - 1.00)	0.045
CPC 1	9 (6.50 - 13.00)	12 (8.00 - 18.00)	0.95 (0.90 - 0.99)	0.018
**Time until airway device insertion**				
Survival	5 (3.00 - 9.00)	6 (4.00 - 10.00)	0.98 (0.93 - 1.02)	0.374
Sustained ROSC	5 (3.00 - 11.00)	6 (4.00 - 9.00)	1.01 (0.99 - 1.05)	0.340
CPC 1 - 2	5 (3.00 - 9.00)	6 (4.00 - 10.00)	0.99 (0.94 - 1.03)	0.553
CPC 1	5 (3.25 - 9.75)	6 (4.00 - 10.00)	0.99 (0.94 - 1.03)	0.643

Supplemental Table S2: The relationship between study endpoints and the time until the initiation of intra-arrest cooling and the time until successful airway device insertion, respectively. These results were obtained using univariate logistic regression in the unmatched dataset. Abbreviations: CPC = Cerebral Performance Category, OR = Odds ratio, CI = Confidence interval, Q1 = First quartile, Q3 = Third quartile.
